# Onset of Type 1 Diabetes Mellitus Five Months After Pembrolizumab Therapy for Nodular Melanoma: A Case Report

**DOI:** 10.7759/cureus.80083

**Published:** 2025-03-05

**Authors:** Eric Wah Sanji, Divine Besong Arrey Agbor, Mary Orians, David Portnoy

**Affiliations:** 1 Internal Medicine, Magnolia Regional Health Center, Corinth, USA; 2 Internal Medicine, Richmond University Medical Center, New York, USA; 3 Oncology, West Cancer Center and Research Institute, Memphis, USA

**Keywords:** diabetic ketoacidosis, immune checkpoint inhibitor, melanoma, pembrolizumab, type 1 diabetes mellitus

## Abstract

Pembrolizumab and other immune checkpoint inhibitors (ICIs) have revolutionized the treatment of melanoma. Nonetheless, they have been linked to endocrinopathies and other immune-related adverse events (irAEs). A rare but potentially fatal side effect, ICI-induced type 1 diabetes mellitus (ICI-DM), usually develops during or soon after therapy. We underscore the significance of long-term monitoring for irAEs by presenting a case of delayed-onset ICI-DM, discovered five months after stopping pembrolizumab.

Our case involves a 47-year-old male diagnosed with nodular melanoma (Breslow thickness, 7 mm; mitotic index, 3), who received adjuvant pembrolizumab for 12 months. Five months after completing therapy, he presented with polyuria and polydipsia and was diagnosed with diabetic ketoacidosis (DKA). Laboratory findings included an HbA1c of 8.3%, low C-peptide (0.49 ng/mL), and negative diabetes autoantibodies. He was diagnosed with pembrolizumab-induced type 1 diabetes and managed with IV insulin in the hospital before being discharged on basal insulin (Tresiba). This case highlights the risk of delayed-onset ICI-DM, even after ICI discontinuation. Clinicians should maintain a high index of suspicion for metabolic complications in ICI-treated patients and provide lifelong monitoring for diabetes.

## Introduction

Pembrolizumab, a PD-1 inhibitor, and other immune checkpoint inhibitors (ICIs) have significantly impacted metastatic melanoma treatment. ICIs enhance T-cell-mediated anti-tumor immunity, drastically improving survival rates. However, immune stimulation can potentially harm healthy organs, causing immune-related adverse outcomes. Endocrinopathies, such as thyroiditis, hypophysitis, and hyperglycemia, are common side effects [1].

ICI-induced type 1 diabetes mellitus (ICI-DM) is a rare but serious disorder with an estimated incidence of 0.2%-1% [2]. It typically requires insulin therapy. The syndrome is characterized by diabetes, low C-peptide levels, and positive autoantibodies (e.g., GAD-65, IA-2) [3]. Although delayed-onset ICI-DM has been observed, most instances occur during or shortly after ICI therapy [4]. Developing type 1 diabetes mellitus (T1DM) following therapy is rare. Typical ICI-DM manifests suddenly, frequently within weeks of initiating ICIs, characterized by severe hyperglycemia or diabetic ketoacidosis (DKA), positive autoantibodies, and immediate insulin dependence resulting from rapid beta-cell destruction. Atypical ICI-DM is characterized by a delayed onset, often taking months or longer. It may present with mild hyperglycemia without DKA, typically lacks autoantibodies, and may not necessitate immediate insulin therapy, indicating a more gradual decline of beta-cells.

We present a case of delayed-onset ICI-DM, detected five months after pembrolizumab discontinuation, emphasizing the need for extended monitoring of immune-related adverse events (irAEs).

## Case presentation

**Figure 1:**
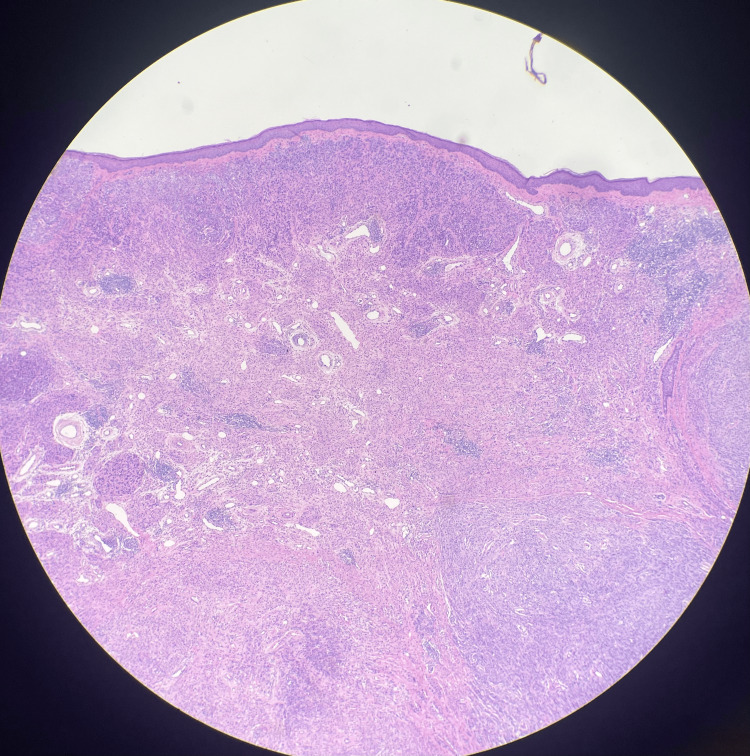
Histologic imaging of nodular malignant melanoma of the left superior helix

A 47-year-old male with no relevant history of T1DM presented to his dermatologist with a lesion on the left superior helix of the ear, initially thought to be a keloid. Over time, the lesion increased in size, prompting a biopsy that revealed nodular melanoma, with a Breslow thickness of 7 mm and a mitotic index of 3, as shown in Figure 1.

A brain MRI showed no evidence of metastasis. The patient underwent wide local excision with clear margins and sentinel lymph node biopsy, which was negative for metastasis. Adjuvant therapy with pembrolizumab (200 mg IV every three weeks) was initiated and continued for 12 months. He tolerated treatment well, with no immediate irAEs reported.

Five months after completing pembrolizumab, the patient developed polyuria, polydipsia, and fatigue of two weeks' duration. Due to worsening symptoms now associated with a two-day history of nausea, vomiting, and abdominal pain, the patient decided to present to the emergency department. Worth noting is that his last A1C prior to these symptoms was 5.2. His laboratory values are shown in Table 1.

**Table 1:** Laboratory values of the patient

Test	Patient Value	Reference Range
Hemoglobin A1c (A1c)	8.30%	<5.7% (normal)
Random Blood Sugar	480 mg/dL	<200 mg/dL
C-Peptide	0.49 ng/mL	0.8-3.85 ng/mL
Serum Ketones	Positive	Negative
Venous pH	7.2	7.35-7.45
Bicarbonate (HCO_3_)	6 mmol/L	22-28 mmol/L
Sodium	139 mmol/L	135-145 mmol/L
Potassium	4.9 mmol/L	3.5-5.0 mmol/L
Thyroid-Stimulating Hormone	1.3 mIU/L	0.4-4.0 mIU/L
Creatinine	0.89 mg/dL	0.6-1.2 mg/dL
Alanine Aminotransferase (ALT)	12 U/L	7-56 U/L
Aspartate Aminotransferase (AST)	13 U/L	10-40 U/L
Total Cholesterol	79 mg/dL	<200 mg/dL
High-Density Lipoprotein (HDL)	40 mg/dL	>40 mg/dL
Low-Density Lipoprotein (LDL)	34 mg/dL	<100 mg/dL
GAD Antibodies	Negative	Negative
IA-2 Antibodies	Negative	Negative

The patient was diagnosed with pembrolizumab-induced T1DM and DKA. He was admitted to the intensive care unit for IV fluids, insulin infusion, and electrolyte correction. After stabilization, he was transitioned to basal insulin (Tresiba, 10 units daily) and discharged with diabetes education and close outpatient follow-up.

At a three-month follow-up, the patient’s glycemic control improved, with an HbA1c of 7.1%. He remained asymptomatic, with no recurrent hyperglycemic episodes.

## Discussion

ICI-DM is thought to result from T-cell-mediated destruction of pancreatic beta cells, akin to classic T1DM. Pembrolizumab, an anti-PD-1 agent, disrupts immune tolerance by inhibiting the PD-1/PD-L1 pathway, potentially leading to the autoimmune destruction of beta cells [5]. The absence of diabetes autoantibodies in this case suggests alternative mechanisms, such as direct T-cell cytotoxicity or cytokine-mediated beta-cell dysfunction [6].

The onset of diabetes five months after pembrolizumab discontinuation is unusual, as most cases occur during or shortly after treatment. This delayed presentation highlights the need for prolonged monitoring of irAEs, even after ICI cessation. Proposed mechanisms for delayed irAEs include persistent immune activation or gradual beta-cell loss [7]. This case contributes to the growing body of literature on the diverse ways ICIs can induce DM. ICI-DM typically presents as T1DM-like, with low C-peptide (as seen in this case) and positive autoantibodies, though atypical cases are reported [3]. The absence of classic T1DM features (e.g., autoantibodies) complicates diagnosis. Based on my literature search, only one case of T1DM was reported seven months after pembrolizumab completion [8].

The primary treatment for ICI-DM involves initiating insulin therapy, as beta cells are typically irreversibly lost. Unlike other irAEs, the administration of immunosuppressive agents, including corticosteroids, has not been effective in reversing ICI-DM. Consequently, patients frequently require lifelong insulin replacement [9]. Insulin degludec was administered as part of a basal insulin regimen to control the patient's blood sugar, in conjunction with oral metformin and empagliflozin. Lifelong glucose monitoring is essential, as beta-cell function may continue to decline over time [10].

## Conclusions

This case highlights the possibility of delayed-onset ICI-DM, even following the cessation of ICIs. Patients treated with ICIs should be monitored closely, by doing frequent blood glucose checks to ensure early diagnosis of ICI-DM. Additional research is required to clarify the mechanisms associated with delayed irAEs and to enhance management strategies.
